# Interleukin-23 as a therapeutic target for inflammatory myopathy

**DOI:** 10.1038/s41598-018-23539-4

**Published:** 2018-04-03

**Authors:** Natsuka Umezawa, Kimito Kawahata, Fumitaka Mizoguchi, Naoki Kimura, Yoko Yoshihashi-Nakazato, Nobuyuki Miyasaka, Hitoshi Kohsaka

**Affiliations:** Department of Rheumatology, Graduate School of Medical and Dental Sciences, Tokyo Medical and Dental University (TMDU), Tokyo, Japan

## Abstract

Current treatments of polymyositis and dermatomyositis (PM/DM) depend on non-specific immunosuppressants. This study was performed to elucidate the role of interleukin (IL)-23, as their possible therapeutic target. As was reported earlier in PM/DM patients, serum IL-23 levels were elevated in mice with C protein induced-myositis (CIM), a murine model of PM. IL-23 was expressed by macrophages in the PM/DM and CIM muscles and by dendritic cells and macrophages in the lymph nodes from the CIM mice. It was also expressed by macrophages in the chemically injured muscles, but not those recruited into the muscles by footpad injection of Freund’s complete adjuvant, demonstrating that IL-23 production should be associated with muscle damage. Genetic deletion of IL-23 as well as preventive and therapeutic administration of blocking antibodies against IL-23p19 subunit suppressed CIM. When lymph node cells from the CIM mice were transferred adoptively into naive wild type or IL-23p19 deficient recipient mice, both recipients developed myositis equally. Thus, elevated IL-23 should promote dendritic cells and macrophages to activate the autoaggressive T cells. Our findings suggest that IL-23 should mediate positive feedback loop from the muscle damage to the T cell activation and be a promising therapeutic target for autoimmune myositis.

## Introduction

Polymyositis (PM) is a chronic inflammatory myopathy that impairs muscle functions to restrict daily activities of the affected patients. Because its precise pathogenesis remains unclear, the standard treatment depends on non-specific immunosuppressants including glucocorticoids and other immunosuppressive agents. Patients under these agents often suffer from their adverse effects and occasionally fail to respond for complete control of the disease activities^1^.

Activated cytotoxic CD8 + T cells, which circulate systemically in patients with PM, play a crucial role in its pathogenesis^2,3^. However, magnetic resonance imaging of the PM muscles demonstrates a patchy pattern consisting of the inflamed and intact muscles. This involvement bias implies that not only autoreactive CD8 + T cells but also local conditioning of the muscles should be required for the myositis development. In C protein-induced myositis (CIM), a murine model of PM^4^, muscle injury is mediated by C protein-reactive CD8 + T cells^5^. In addition, the activated T cells could induce transferred myositis only in the muscles where the local innate immunity was activated with footpad injection of Freund’s complete adjuvant (CFA)^6^. We thus proposed “seed and soil” model of autoimmunity; “seed” stands for the autoreactive T cells while “soil” for the target tissues. Both have to be activated for the development of autoimmune myositis.

Our previous report disclosed that recruited macrophages into the muscle in response to the footpad CFA injection could not develop myositis on their own, but are responsible for “soil” activation by producing Interleukin (IL)-1 and tumor-necrosis factor alpha^6^. However, these cytokines are also expressed by muscle fibers during homeostatic regeneration^7^ and unpromising as therapeutic targets for PM/ dermatomyositis (DM) in clinical settings^8^. Thus, as a therapeutic target of PM/DM, we should explore specific molecules expressed in the damaged muscles.

It was reported that IL-23 was higher in sera from PM/DM patients than in those from healthy donors^9,10^, and expressed by macrophages and dendritic cells in the PM/DM muscles^11^. Although these facts indicate its pathological contribution to PM/DM, little has been known about its roles in inflammatory myopathy.

IL-23 is a member of IL-12 cytokine family^12,13^ and consists of IL-23 subunit p19 (IL-23p19) and IL-12 subunit p40 (IL-12p40). The IL-23R and IL-12Rβ1 subunits comprise the IL-23 receptor complex and bind to IL-23p19 and IL-12p40, respectively^14^. IL-23 is produced primarily by activated macrophages and dendritic cells (DCs). It expands Th17 cells and maintains their phenotype such as their cytokine production including IL-17A, which is their major effector molecule^15^. Besides the roles on Th17 cells, IL-23 acts on macrophages and DCs to promote antigen presentation and proinflammatory cytokine production^16^. This fact does not necessarily warrant little involvement of IL-23 in autoimmune myositis although IL-17A is dispensable for CIM^17^. Since IL-23 bridges innate and adoptive immunity, it could play a pathological role in myositis.

In the present study, we found that IL-23 is expressed in the damaged muscles. To reveal its pathological involvements, the effects of IL-23p19 deficiency and IL-23 blockade with anti-IL-23 receptor antibodies on CIM were studied. The adoptive transfer of CIM was performed to discern whether IL-23 is involved in “seed” and/or “soil” activation in the pathogenesis. We propose a novel therapeutic approach for inflammatory myopathy targeting IL-23.

## Results

### The serum concentrations of IL-23 were elevated in CIM mice

**Figure 1:**
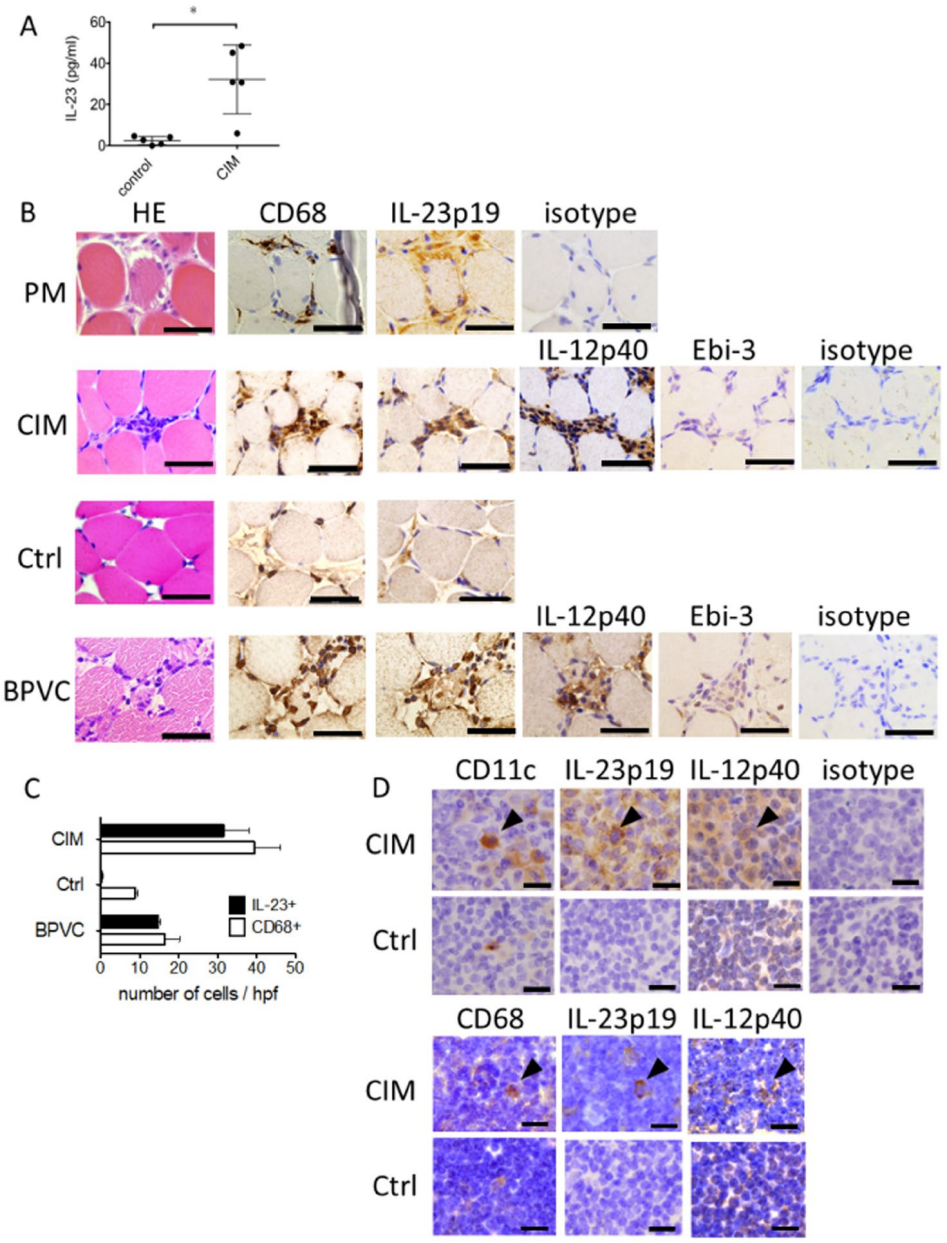
Expression of IL-23 in the sera, muscles and lymph nodes. (**A**) Concentrations of IL-23 in the sera from CIM mice (CIM) (n = 5) and controls (n = 5) measured with specific ELISA. Unimmunized mice treated with CFA alone were employed as controls. Bars represent the mean and standard deviation of the IL-23 concentrations. An asterisk represents statistically significant difference (p < 0.05). (**B**) HE staining and immunohistochemical staining for IL-23p19, CD68 and IL-12p40 of the serial sections of muscle specimens from a PM patient (PM), CIM mice (CIM), control mice with their footpads treated with CFA (Ctrl) and BPVC-treated mice (BPVC). Representative figures of staining with polyclonal rabbit IgG were shown (isotype). Staining of the muscles from other PM/DM patients represented the similar findings. Scale bars show 50 μm. (**C**) Number of IL-23 + (filled box) and CD68 + (blank box) cells in the muscle specimens from the CIM mouse (CIM), control (Ctrl) and BPVC-treated mouse (BPVC). Representative 4 high power fields were evaluated for each group. Boxes represent mean of the 4 fields and the error bars represent standard deviation. (**D**) Immunohistochemical staining for IL-23p19, IL-12p40, CD11c and CD68 of inguinal lymph nodes (LNs) from CIM mice (CIM) and control mice with their footpads treated with CFA (Ctrl). Representative figures of staining with polyclonal rabbit IgG were shown (isotype). Arrow heads show presentative CD11c + /IL-23p19 + /IL-12p40 + and CD68 + /IL-23p19 + /IL-12p40 + cells. Scale bars show 5 μm.

As was stated earlier, patients with active PM/DM had higher concentrations of IL-23 in sera than healthy donors^9,10^. To study if this is the case with the CIM mice, we examined serum from the CIM mice for concentrations of IL-23p19/IL-12p40 complexes 14 days after the immunization. The CIM mice had significantly more IL-23 in the sera than control mice treated at the footpads with CFA alone (Fig. 1A). Thus, non-specific immune activation with adjuvant was not responsible for the elevated IL-23.

### IL-23p19 was expressed by CD68 + cells at the endomysium of the PM/DM and CIM muscles

To locate the IL-23 expressing cells, we examined the muscles from the PM/DM patients and CIM mice immunohistochemically for IL-23p19 expression. CD68 + mononuclear cells infiltrating into the affected muscles of PM/DM patients expressed IL-23p19 (Fig. 1B), confirming the notion in the previous report^11^ with antigen specificity. In CIM mice, CD68 + cells in the muscles expressed IL-23p19 and IL-12p40 14 days after the immunization (Fig. 1B). Muscle fibers did not express IL-23p19 or IL-12p40. Since IL-23p19 is shared with IL-39, we studied the expression of Epstein-Barr virus-induced 3 (Ebi3), which is the other subunit of IL-39^13^. IL-23p19 + cells in the muscles did not express Ebi-3 (Fig. 1B). These results indicated that CD68 + cells expressed IL-23, but not IL-39.

As a control, we treated mice with footpad injection of CFA without C protein-fragments. While this treatment promotes macrophages to accumulate into the muscles of the ipsilateral leg without the muscle damages^6^, the accumulated CD68 + in the muscles did not express IL-23p19 (Fig. 1B).

Since the above results implied that muscle damage should be associated with IL-23p19 expression, we studied the muscles treated with intramuscular injection of bupivacine hydrochloride (BPVC) to induce chemical damage. As was reported previously^7^, this injection induced muscle necrosis and subsequent infiltration of mononuclear cells, which were apparent 2 days after the treatment (Fig. 1B). As is in the case of the CIM muscles, CD68 + cells in the chemically injured muscles were positive for IL-23p19 and IL-12p40, but not Ebi-3 (Fig. 1B).

In terms of the number, CD68 + cells increased in the muscles from the CIM mice, mice treated with CFA alone and BPVC-treated mice. In contrast, IL-23p19 + cells were found only in the CIM and BPVC-treated mice (Fig. 1C).

### IL-23p19 was also upregulated in draining lymph nodes from CIM mice

Because DCs and macrophages, which participate in antigen presentation at LNs, could be the source of IL-23, we examined the inguinal LNs for IL-23, CD11c and CD68 expression immunohistochemically. The inguinal LNs are the draining LN of the inflamed muscles of CIM mice and those of the mice treated with the footpad CFA injection. Expression of both IL-23p19 and IL-12p40 by CD11c + cells at the paracortex and medulla or CD68 + cells at the cortex was upregulated in the CIM mice compared to the mice treated with CFA alone (Fig. 1D).

### IL-23 was essential for the development of CIM

**Figure 2:**
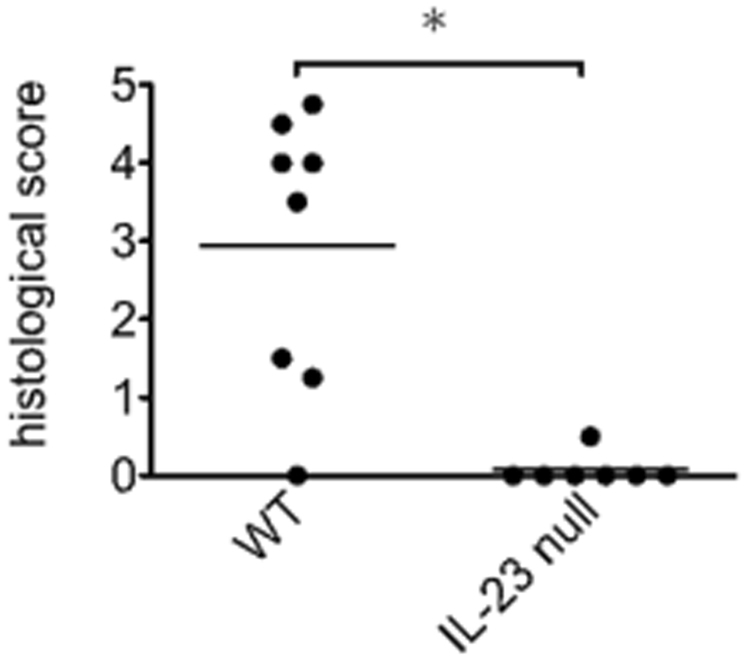
Histological scores of myositis in IL-23p19 null mice and WT mice with CIM. The muscle specimens from 8 wild-type (WT) and 7 IL-23p19 null (IL-23 null) mice were examined histologically 14 days after the immunization for the severity of CIM. The incidences of CIM in the two groups were 87% and 14%, respectively. The results are representative of 2 independent experiments. Bars show the mean of the histological scores. An asterisk represents statistically significant difference (p < 0.05).

To discern if IL-23 is required for development of CIM, IL-23p19-null mice were immunized with the C protein fragments. The muscles were evaluated histologically 14 days after the immunization. The severity of myositis was suppressed significantly in IL-23p19-null mice compared to the wild-type (WT) (p < 0.05) (Fig. 2).

**Figure 3:**
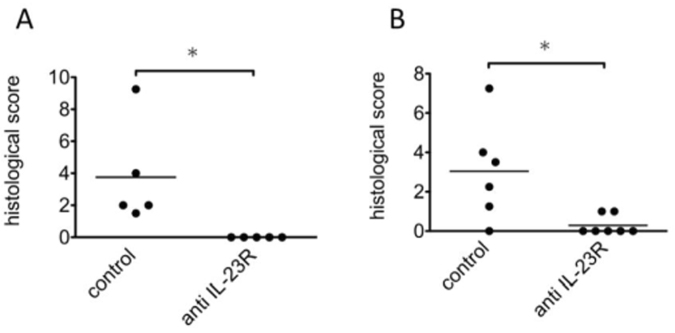
Effects of anti-IL-23R monoclonal antibodies on CIM. (**A**) Preventive treatment of WT mice with CIM with control antibodies (control) and anti-IL-23R monoclonal antibodies (anti IL-23R). The antibodies (1.0 mg/mouse) were administrated intraperitoneally a day before and 6 days after the immunization. The incidences of CIM in the two groups were 100% and 0%, respectively. (**B**) Therapeutic treatment of WT mice with CIM with the same dose of the antibodies 7 and 11 days after the immunization. The severity of CIM was evaluated histologically 14 days after the immunization. The incidences of CIM in the two groups were 83% and 28%, respectively. The results are representative of 2 independent experiments. Bars show the mean of the histological scores. Asterisks represent statistically significant differences (p < 0.05).

Next, we examined the effects of the IL-23 blockade on CIM by administration of blocking antibodies against IL-23R. When mice were subjected to anti-IL-23R antibody administration from the day before the immunization, the incidence and severity of CIM were suppressed significantly in the treated group (p < 0.05) (Fig. 3A). These results were in accordance with the results of the IL-23p19-null mice, and demonstrated that IL-23 should play a crucial role in the development of CIM.

### Administration of anti-IL-23R antibodies after the onset of the myositis ameliorated CIM

To discern whether IL-23 blockade could be a therapeutic strategy for autoimmune myositis, the anti-IL-23R antibodies were administrated to CIM mice in a therapeutic protocol. More precisely, treatments were started from 7 days after the immunization, when myositis is evident histologically. In comparison with mice treated with the control antibodies, the severity of the myositis was suppressed significantly in mice treated with the anti-IL-23R antibodies (p < 0.05) (Fig. 3B).

### Local conditioning of the muscles was preserved in IL-23p19-null mice

**Figure 4:**
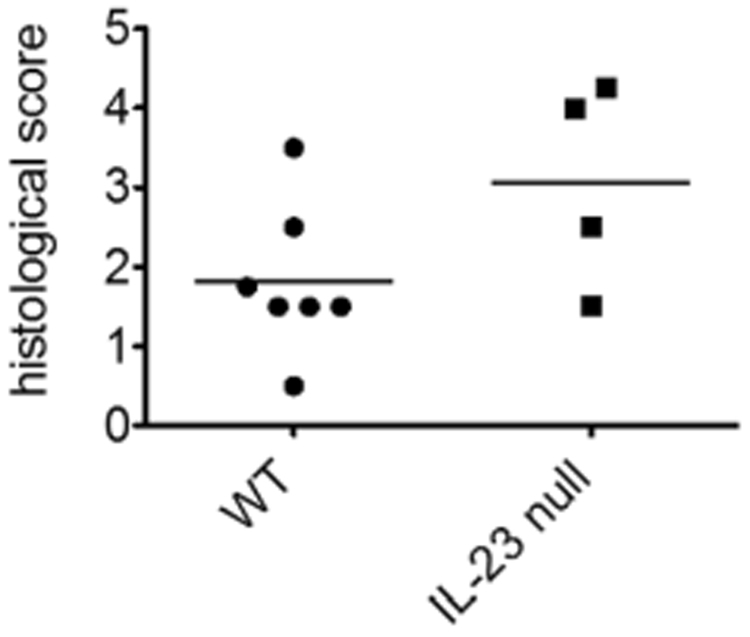
Adoptive transfer of CIM. LN cells from WT mice with CIM were stimulated with recombinant IL-2 and BMDCs that were pulsed with C protein fragments. After 3-day co-culture, the non-adherent cells were harvested and transferred into 7 wild-type (WT) or 4 IL-23p19 null mice (IL-23 null) recipients with their footpads treated with CFA. The severity of myositis in the recipients was evaluated histologically 14 days after the adoptive transfer. All recipient mice in both groups developed adoptively transferred myositis. The results are representative of 2 independent experiments. Bars show the mean of the histological scores.

As IL-23 acts in both acquired and innate immunity, it could contribute to the pathological processes of “seed” and/or “soil” in CIM. To elucidate the target of the IL-23 blocking treatments, we performed adoptive transfer of CIM to IL-23p19-null recipients. LN cells from CIM mice were stimulated with C protein fragment-pulsed bone marrow-derived dendritic cells (BMDCs) for 3 days, and were transferred into IL-23-null or WT recipient mice with their footpads treated with intradermal injection of CFA. The severity and incidence of myositis in the IL-23p19-null recipients were comparable to those in the WT recipients (Fig. 4). If IL-23 acts mainly on the “soil” activation to allow the T cells attack, transferred myositis in IL-23p19 recipients should be less severe than in WT mice. Thus, IL-23p19 should have little contribution to the local conditioning of the muscles.

## Discussion

We found that IL-23 is produced by CD68 + macrophages in the damaged muscles and by CD11c + DCs and CD68 + macrophages in the draining LN from CIM mice. Since its blockade ameliorated CIM therapeutically, IL-23-targeting therapy should be a new approach to treat PM/DM.

IL-23 was not expressed in the muscles and in LN from CFA-treated mice without the C protein-immunization. Thus, IL-23 upregulation does not result from non-specific inflammation but from pathological processes intrinsic to the muscle damage. Since the IL-23 upregulation in CD68 + macrophages was observed in the chemically injured muscles, unspecified factors from the damaged muscle fibers, such as damage-associated molecular patterns, should be responsible for the IL-23 production in the muscles and LNs.

While the IL-23p19-null mice were resistant to CIM, they were susceptible as recipients to the adoptive transfer of CIM. We recognize that the transferred cells contained not only activated CD8 + T cells but also stimulated DCs that could potentially activate “soil” in the recipients. However, we reported that the transferring donor cells alone is not enough to activate “soil” to induce myositis in the transfer model^6^. Taken together, our results suggested the role of IL-23 not on the “soil”, but on “seed” activation.

Considering the serum IL-23 levels and IL-23 expression in the draining LN were upregulated in the CIM mice, it is likely that IL-23, which had been induced by preceding damage of the muscles, should augment systemic acquired immunity involved in the CIM. Thus, IL-23 should mediate a positive feedback loop of myositis, connecting the muscle damage to further T cell activation.

Since DCs rather than macrophages should be important in antigen presentation, IL-23 from macrophages should act on DCs to accelerate T cell priming in a paracrine manner while IL-23 from DCs should act in autocrine and paracrine manners. This notion is supported by two reports on the effects of IL-23 on murine CD8 + DCs^18,19^. While IL-23 activates CD8 + and CD8- DCs to potentiate antigen presentation and cytokine production, only CD8 + DCs can cross-present the extracellular antigens to CD8 + T cells. Since CIM is a CD8 + T cell-mediated model^5^, the effects of IL-23 on CD8 + DCs should be important for myositis development. In contrast to IL-23, IL-12, a structural and functional analogue of IL-23, acts selectively on CD8- DCs^19^ and should not be able to substitute for IL-23. In humans, IL-23 might act on the functionally analogous DCs to activate pathological T cells in PM/DM.

IL-23 acts also on retinoic acid receptor-related orphan receptor-γt + T cells including Th17 cells^13,20^ but not on CD8 + T cells^14,15,21^. In most of autoimmune disease models of mice, IL-23 exerts its effects primarily through expansion of the Th17 cells that produce IL-17A as an effector molecule^22^. However, suppression of this IL-23/17 A axis should not be responsible for the effects of the IL-23 blockade in CIM since IL-17Ais dispensable for the CIM development^17^. It is noteworthy that IL-23 blockade, but not IL-17 blockade was beneficial in Crohn’s disease (CD)^23^. Since IL-23 is expressed by macrophages in the gut from the affected patients^24^, pathological contribution of IL-23 might be shared by CD and autoimmune myositis.

Because Th17 cells are absent in IL-23p19-null mice^25^, it is possible that other cytokines secreted from Th17 cells than IL-17A might contribute to the pathogenesis of CIM. They include IL-17F, IL-21 and IL-22, but have not been suggested as activators of CD8 + T cells^26^. Experimental autoimmune encephalomyelitis, a representative Th17 cell- dependent model of multiple sclerosis, required IL-17A but not the other Th17 cytokines for the disease development^27–29^. It is thus likely that Th17-related cytokines should have little effect on CIM.

It was reported that IL-23 expression is upregulated in various kinds of human tumors^30^. Excessive supply of IL-23 from tumors was suggested to activate tumor-specific CD8 + T cells in mice and humans^31,32^. Stimulation of muscle-reactive CD8 + T cells by tumor-derived IL-23 might be background for the higher incidence of tumors in PM/DM than general populations^33^. In this regard, tumor removal might be effective for reducing activities of PM/DM especially in the cases with IL-23-producing tumors.

Since muscle weakness is a primary feature of PM/DM, we^4,7^ and others^34^ evaluated muscle strength of the CIM mice with grip and rotarod tests. However, the results can depend on the attention of the individual mice and are often unreproducible. Serum levels of muscle-derived enzymes were irrelevant to the severity of CIM, probably because they varied depending on their individual physical activities. At the moment, the histological scoring is the most reliable way to assess the severity of CIM^6^.

In conclusion, we demonstrated the significant role of IL-23 in CIM and likely in PM/DM. Notably, the relative safety of IL-23 blockade with ustekinumab has been assured with accumulating evidences in clinical settings^22^. IL-23p19-specific antagonists including guselkumab^35^ and tildrakizumab^36^ are also being tested for treatment of psoriasis, CD and ankylosing spondylitis^22^. These agents would enable us to apply the IL-23 targeting therapy to treatment of PM/DM, and to reveal the clinical significance of IL-23.

## Methods

### Mice

Female C57BL/6 mice were purchased from Charles River Japan (Yokohama, Japan). IL-23p19 null mice^37^ were obtained from RIKEN (Yokohama, Japan). All animal experiments were approved by the Institutional Animal Care and Use Committee of TMDU and were performed in accordance with the institutional and national guidelines.

### Patients and muscle biopsy

Muscle biopsy specimens were obtained from an untreated PM and two untreated DM patients who met the Bohan and Peter criteria and Tanimoto’s criteria of PM/DM^38^. All patients provided their written informed consent. All experiments using the muscle specimens from patients were approved by the institutional review board at TMDU and were performed in accordance with the principles of the Declaration of Helsinki. Written informed consent was obtained from all participants.

### Treatment of mice

Mice were immunized with recombinant C protein fragments, which derived from the human fast-type skeletal muscle, for CIM induction^2^. In brief, recombinant C protein fragments emulsified in CFA were injected subcutaneously on the tail base and footpads of mice. Some mice were injected intraperitoneally with anti-IL-23R monoclonal antibodies (clone 21A4)^39^ or isotype-matched control monoclonal IgG1 (clone 27F11) that were provided by Merck Sharp & Dohme Corp. (Palo Alto, CA). The quadriceps of mice were injected with 50 μl of 0.5% BPVC (Sigma) to provoke muscle injury^7^. For adoptive transfer of CIM, LN cells from the CIM mice and C protein fragment-pulsed BMDCs were cultured with human recombinant IL-2 (Kyowa Pharmaceutical Industry Co., Ltd. Osaka, Japan) for 3 days. Eight million cultured LN cells were transferred to naïve mice with intradermal injection of CFA at their footpads^6^.

### Histological evaluation of myositis

The hematoxylin and eosin (H&E) stained 10 μm sections of the quadriceps and hamstrings were examined in a blinded manner for the presence of mononuclear cell infiltration and degeneration of the muscle fibers. The severity of myositis was graded histologically on the scales of 1–6, where 1 = involvement of 1 muscle fiber, 2 = involvement of 2–5 muscle fibers, 3 = involvement of 6–15 muscle fibers, 4 = involvement of 16–30 muscle fibers, 5 = involvement of 31–100 fibers, 6 = involvement of >100 muscle fibers^7^. When multiple lesions with the same grade were found, 0.5 was added to the grade. The score of each muscle was evaluated by averaging scores of 2 different sections. The histological scores of the individual mice were calculated by summing scores of the quadriceps and hamstrings.

### ELISA and Immunohistochemistry

Serum level of IL-23 was quantified with Quantikine ELISA kit (R&D Systems) according to the manufacturer’s instructions. Formalin-fixed and paraffin-embed sections of muscles were deparaffinized and heated in a pressure pot for 3 min in citrate buffer (10 mM, pH6) to retrieve antigens. Then, the sections were stained with rabbit anti-mouse/human IL-23p19 polyclonal antibodies (Abs) (ab115759; Abcam), mouse anti-human CD68 monoclonal Abs (M0814; DAKO), rat anti-mouse CD68 monoclonal Abs (MCA1957F; AbD Serotec), Armenian Hamster anti-mouse CD11c monoclonal Abs (557400, BD Pharmingen), rabbit anti-mouse IL-12p40 polyclonal Abs (ab106270; Abcam) and rabbit anti-mouse Ebi-3 polyclonal Abs (ab83896; Abcam). Polyclonal rabbit IgG (AB-105C; R&D), mouse IgG1 (MAB002; R&D), rat IgG2a (MAB006; R&D) or Hamster IgG (sc-2864, Santa Cruz) were used as isotype controls. Non-specific staining was blocked with 1% bovine serum albumin (A7906; Sigma) in PBS. The bound antibodies were visualized with peroxidase-labeled amino acid polymer-conjugated goat anti-rabbit IgG (K4002, DAKO), anti-mouse IgG (K4000, DAKO), anti-rat IgG (414311 F, Nichirei Biosciences) or HRP labeled goat anti-Armenian Hamster IgG (sc-2443, Santa Cruz) Abs and the associated substrate, diaminobenzene (K3468, DAKO). The following samples were employed as positive controls for each molecule; mouse kidney for IL-23p19, mouse spleen for IL-12p40/CD68/CD11c and RAJI cells for Ebi-3^23,30,39^.

### Statistical analyses

Histological scores were analyzed statistically with Mann Whitney U test. The concentrations of IL-23 were analyzed with Student t-test.

### Ethical approval

This study was approved by the local institutional review board at Tokyo Medical and Dental University (TMDU), Tokyo, Japan.
